# The Potential Power of Bar-HRM Technology in Herbal Medicine Identification

**DOI:** 10.3389/fpls.2016.00367

**Published:** 2016-03-30

**Authors:** Wei Sun, Jing-jian Li, Chao Xiong, Bo Zhao, Shi-lin Chen

**Affiliations:** ^1^Institute of Chinese Materia Medica China Academy of Chinese Medical SciencesBeijing, China; ^2^College of Forestry and Landscape Architecture South China Agricultural UniversityGuangzhou, China; ^3^Zhuhai College of Jilin UniversityZhuhai, China

**Keywords:** Bar-HRM technology, herbal medicine, adulteration, substitution, drug safety

## Abstract

The substitution of low-cost or adulterated herbal products for high-priced herbs makes it important to be able to identify and trace herbal plant species and their processed products in the drug supply chain. PCR-based methods play an increasing role in monitoring the safety of herbal medicines by detecting adulteration. Recent studies have shown the potential of DNA barcoding combined with high resolution melting (Bar-HRM) analysis in herbal medicine identification. This method involves precisely monitoring the change in fluorescence caused by the release of an intercalating DNA dye from a DNA duplex as it is denatured by a gradual increase in temperature. Since the melting profile depends on the GC content, length, and strand complementarity of the amplification product, Bar-HRM analysis opens up the possibility of detecting single-base variants or species-specific differences in a short region of DNA. This review summarizes key factors affecting Bar-HRM analysis and describes how Bar-HRM is performed. We then discuss advances in Bar-HRM analysis of medicinal plant ingredients (herbal materia medica) as a contribution toward safe and effective herbal medicines.

## Introduction

Herbal medicines have played a significant role in preventing chronic diseases and improving health for human beings since ancient times. According to the World Health Organization [WHO] (2003), over 70% of the population in developing countries uses herbal products (http://www.who.int/mediacentre/news/releases/2004/pr44/en/). The past decade has seen increasing global demand for herbal medicines despite the economic recession (GIA, 2013). Herbal medicines are clearly gaining global influence in modern medical and health services. At an international conference in February 2013, WHO Director General Dr. Margaret Chan stated that traditional medicines of proven quality, safety, and efficacy contribute to the goal of ensuring that all people have access to care (World Health Organization, 2013). Consumer safety is an issue that cannot be overlooked as the herbal industry grows. However, the large number and variety of medicinal plants currently sold in various markets, along with the fact that some of them are commonly sold in processed or modified forms (e.g., dried material, tablets, powders, and capsules), presents a challenge to efforts to accurately distinguish genuine products from their close relatives, inferior substitutes, adulterants, and counterfeits (Newmaster et al., 2013; Veldman et al., 2014). Inaccurate identification of herbal material has led to several safety-related incidents. For example, due to their similar appearance and Chinese names, a herbal ingredient derived from *Astragalus complanatus* bunge (shayanzi), which is used for diabetes treatment, was inadvertently replaced with one from *Hyoscyamus niger* (tianxianzi), which interrupts the parasympathetic nervous system. *H. niger* contains L-Hyoscyamine Atropine and Scopolamine, natural compounds derived from *Hyoscyamus* L. species, which can overstimulate the sympathetic nervous system and cause convulsions and, eventually, death (Li et al., 2012; Tu et al., 2014). Incidents involving the use of Radix Stephaniae tetrandrae (Fangji), derived from *Stephania tetrandra* S. Moore, have also been reported (Vanherweghem et al., 1993; Grollman et al., 2007; Debelle et al., 2008; Poon et al., 2013). In one case, patients were prescribed the Chinese herb Fangji (*Stephania tetrandra* S. Moore) for weight loss, but were instead given Guangfangji (*Aristolochia fangchi* Y. C. Wu ex L. D. Chow and S. M. Hwang), which contains aristolochic acids. The patients subsequently developed aristolochic acid nephropathy (Lord et al., 1999). Despite this, the marketing of herbs containing Aristolochiaceous materials continues in China (Wu et al., 2015). In addition to safety concerns, the quality of herbal products has been a subject of increasing attention. Although commercial herbal products have to be authentic as well as safe, adulterated herbal materials have been found on sale (Ouarghidi et al., 2012; Xin et al., 2015). For example, processed materials of the relatively rare herbaceous perennial *Panax ginseng* C. A. Mey are commonly adulterated with material from the low-cost herb *Panax quinquefolius* (Chen et al., 2008). Accurate identification of medicinal plant ingredients is therefore important to safeguard customer health and ensure the quality and authenticity of herbal products.

Various criteria and methods have been developed to authenticate medicinal plant ingredients. Traditional methods include the use of morphological characteristics, microscopy, and chemical profiles (Li et al., 2010; Lau et al., 2012; Shen et al., 2014), and these methods have played a major role in herbal drug authentication and quality control since the birth of pharmacognosy. However, these techniques all have limitations. In addition to possible confusion caused by morphological similarity or variation in chemical profiles between samples, the accuracy of these methods depends on the expertise of the assessor (Li et al., 2011). In addition, morphological identification is often not possible when the original plant material has been heavily processed. Additional methods have therefore been sought. Advancements in molecular biology and molecular genetics have allowed the application of DNA-based methods to identify and authenticate medicinal plants. DNA barcoding combined with high resolution melting (Bar-HRM) analysis is a novel, advanced method which has recently been successfully applied in herbal medicine authentication (Mader et al., 2011; Bosmali et al., 2012; Xanthopoulou et al., 2016).

In this review, we introduce the principle of HRM, point out key factors affecting the analysis, and describe how HRM is performed and how the data analyzed. We then continue with a brief overview of its current role in scientific studies, and finally present and critically discuss the potential power of Bar-HRM analysis based methods in herbal medicine authentication.

## High Resolution Melting

### Overview of HRM Technology

**FIGURE 1:**
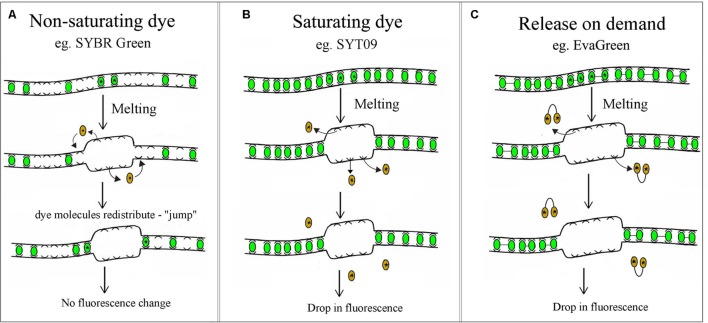
**Non-saturating, saturating and “release-on-demand” dsDNA intercalating dyes.** **(A)** At non-saturating concentrations the dye quickly rebinds to regions that remain double stranded, consequently there is no drop in fluorescence. **(B,C)** Saturating and “release-on-demand” dyes do not redistribute from the melted regions of single-stranded DNA back to dsDNA, resulting in a reduction of fluorescence. This difference gives dyes such as SYT09 or EvaGreen the high sensitivity required for HRM analysis.

High Resolution Melting technology characterizes nucleic acid samples based on their disassociation behavior, using direct melting to detect small sequence differences in PCR-amplified sequences. These differences are detected through the use of DNA-specific dyes, high-end instrumentation and sophisticated analysis software. Samples are discriminated according to their composition, length, GC content, and strand complementarity (Ririe et al., 1997; Reed and Wittwer, 2004; Palais et al., 2005). The first step is standard PCR amplification of the region of interest in the presence of a specialized double-stranded DNA (dsDNA) binding dye (Wittwer et al., 1997, 2003; Herrmann et al., 2006). There are various types of dsDNA intercalating dyes, including SYBR Green, LC Green PLUS, Eva Green, SYTO9, and ResoLight. SYBR Green is the most common non-saturating dsDNA intercalating dye. It is generally unsuitable for most HRM applications because it has been shown to inhibit PCR at high concentrations and has been hypothesized to redistribute from melted regions back into the dsDNA amplicon, as detailed in **Figure 1A** (von Ahsen et al., 2001; Graham et al., 2005). To overcome this limitation, a new class of dsDNA intercalating dyes named saturating or “release-on-demand” dyes that do not inhibit DNA polymerases, or alter the Tm of the product, have recently been developed. In contrast to SYBR Green, Saturating dyes such as SYTO9 or LCGreen do not inhibit the DNA polymerase at rather high concentrations, ensuring more complete intercalation of the amplicon. More precise examination of the melting behavior is therefore possible for HRM analysis, as indicated in **Figure 1B** (Vossen et al., 2009). The “release-on-demand” dyes, e.g., EvaGreen, can be used at non-saturating concentrations. Due to its novel mechanism of fluorescence emission, the fluorescent signal is quenched when the dye is free in solution. Instead, the dye emits high fluorescent signal when it binding to duplex DNA (**Figure 1C**). There is no PCR inhibition, whilst the unique dye provides highly sensitive HRM analysis.

**FIGURE 2:**
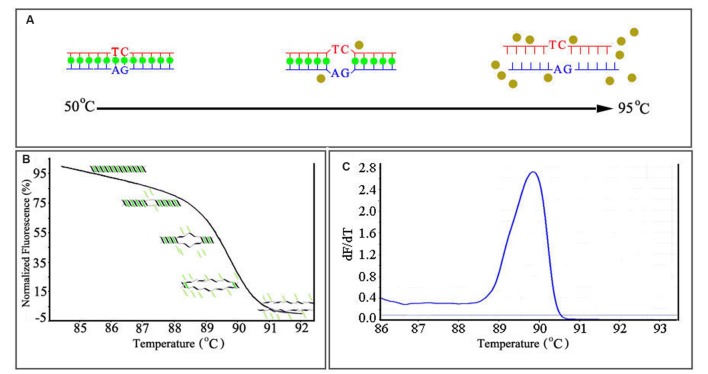
**The mechanism of real-time HRM system.** **(A)** The dissociation-characteristics (Melting) of double-stranded DNA during heating. **(B)** The normalized plot generated by high resolution melting (HRM) analysis. High fluorescence when dye is bound to dsDNA. **(C)** The melting curves (derivative melt curves) of the amplicons, negative derivative of the fluorescence (F) over temperature (T) (–dF/dT) against the temperature (T).

As mentioned above, the saturating or “release-on-demand” dyes have high fluorescence when bound to dsDNA and low fluorescence when unbound, allowing the user to monitor DNA amplification during PCR (**Figure 2A**). HRM analysis begins after PCR amplification. The amplicon is gradually heated from around 50°C to around 95°C; the gradual denaturation resulting from incremental heating produces a characteristic melting profile (Wittwer et al., 2003). When the dsDNA dissociates into single strands, the intercalating dye is released and fluoresces at a low level. The change in fluorescence is plotted against the temperature, generating a melting curve (**Figure 2B**) characteristic of the amplicon. The melting temperature (Tm) of the amplicon can be determined from the peak obtained by plotting the negative derivative of the fluorescence (F) over temperature (T) (–dF/dT) against the temperature (T; **Figure 2C**) (Montgomery et al., 2007). Since different genetic sequences melt at slightly different rates, they can be viewed, compared, and detected using these curves. When correctly set up, HRM is sensitive enough to allow the detection of a single base change between otherwise identical nucleotide sequences (Wittwer et al., 2003; Zhou et al., 2005; Krypuy et al., 2006).

### Key Factors Affecting HRM Assays

For successful analysis, care should be taken to ensure that the experiment is optimized for HRM. Particular attention should be given to primer design, PCR reagents and cycling conditions, since small differences in melting curves can arise from sources other than the nucleotide sequence. Factors such as genomic DNA (gDNA) quality, amplicon length, primer design, dye selection, and PCR conditions will all affect the melting behavior (Montgomery et al., 2007; van der Stoep et al., 2009). Achieving specific amplification is critical to the success of the assay, since any non-specific amplification will greatly impair the melt analysis. For example, a low-quality DNA template may produce non-specific PCR products, resulting in failed reactions or low sensitivity and incorrect genotype calls. Samples that amplify late or fail to reach a high signal plateau in the PCR phase can lead to inconclusive or low-resolution HRM data. For the best results, all DNA samples in an analysis run should be prepared using the same method. DNA samples should not differ significantly in their concentration in order to ensure similar cycle threshold (Ct) values (Reed et al., 2007; Vossen et al., 2009). Furthermore, HRM analysis should be performed immediately following PCR whenever possible. An amplicon length of 50–300 bp is generally recommended for HRM analysis of different sequence variants, including single nucleotide polymorphisms (SNPs), inversions, insertions, and deletions. The longer the amplicon, the more difficult it is to clearly discriminate between sequence variants. However, longer amplicons (typically 200–500 bp) can be used when screening for unknown sequence differences (Montgomery et al., 2007, 2010; Erali et al., 2008). This is useful in gene scanning or determining the variation within a population. Primers designed for HRM analysis should be optimized for robust performance and specificity to the region of interest, since the dyes will bind to any dsDNA products. In addition, HRM software may not be able to detect non-specific reaction products if their melting profiles are similar. It is therefore best to initially assess PCR products by agarose gel electrophoresis (Wittwer, 2009). Other important factors are the PCR conditions and the choice of intercalating dye. The PCR conditions should be optimized in order to achieve efficient amplification, indicated by low *C*t-values and amplification curves plateauing (Vossen et al., 2009). Several different HRM dyes should be tested to determine which functions best in a given experimental system. One factor affecting dye functionality is the instrument itself, since different instruments use different detection methods.

### HRM Data Analysis

**FIGURE 3:**
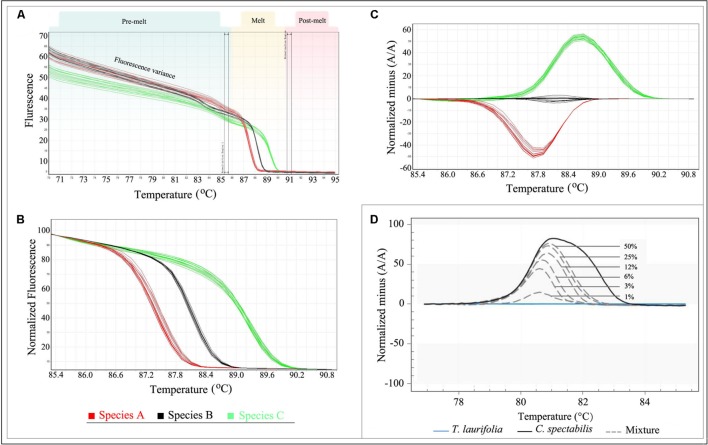
**Analysis of HRM data.** **(A)** Raw Data Melt Curve. **(B)** Normalization Data derived from the raw data plots in **(A)**. **(C)** Difference Graph derived from the Normalization Data. **(D)** Specific amplicons and applied to reference mixtures containing 50, 25, 12, 6, 3, and 1% of *C. spectabilis* in *T. laurifolia*. Note: **Figure 3D** adopted from reference (Singtonat and Osathanunkul, 2015).

With advanced software tools, data analysis is typically straightforward, and multiple samples can be analyzed simultaneously. However, it is important to know what to look for when analyzing raw HRM melting profiles. The raw data collected during HRM analysis includes fluorescence readings across a range of temperatures which consists of three parts: pre-melt, melt, and post-melt (**Figure 3A**). The variance makes it difficult to properly analyze the results even though different genotype groups may be visible. The parallel double-bars in the interface should be positioned to select pre- and post-melt regions with which to normalize data (**Figure 3A**). If the pre- or post-melt regions cannot be clearly identified, the HRM run should be repeated with the temperature range adjusted as required.

When the data is normalized correctly, it will appear as shown in **Figure 3B.** This is termed “Normalization Data.” In this plot, the fluorescence variance of the pre- or post-melt regions has been removed, and only the temperature range between the outer bars of the pre- and post-melt regions is shown. The genotypes are now more distinct, but the differences between melting curves are often small in some cases. In order to better visualize small differences between individual melting curves, some HRM software applications allow calculation of a difference plot (**Figure 3C**). In this plot, sample curves are subtracted from a single reference run in the same experiment. Any genotype can be selected as the reference, but typically a wild-type control is used; in the case of species identification, genotypes of test samples can be defined by selecting a representative sample for each species. In addition, a genotype confidence percentage (GCP; the confidence that a sample is the same as the reference genotype, with a value of 100 indicating an exact match) is calculated for each genotype by some HRM software. Depending on the study’s objective, the confidence threshold can be adjusted to group the samples. However, it is advisable to confirm the genotypes by checking the difference plot.

## Bar-HRM Method to Identify and Certify Herbal Material

High resolution melting analysis has several advantages over traditional methods for gene scanning and genotyping. The analysis is performed immediately after amplification and it is not necessary to purify or separate the amplicons, making HRM analysis particularly suitable for medium- to high-throughput amplification. In addition, HRM analysis is less expensive than other approaches, such as DNA sequencing. Its ease of use, flexibility, low cost, superb sensitivity, and specificity have led to the adoption of HRM for clinical research and diagnostics, including the detection of cancer-specific mutations (Akiyoshi et al., 2013; Lin et al., 2014) and DNA methylation (Wojdacz, 2012; Wojdacz et al., 2014), as well as for the authentication of food products (Ganopoulos et al., 2012; Madesis et al., 2012; Sakaridis et al., 2013) and the accurate quantitation and detection of bacteria (Duyvejonck et al., 2015). Very recently, a new method combining DNA barcoding with HRM analysis (Bar-HRM) was developed for the authentication of herbal medicines and for accurate quantitation of adulterants in commercial herbal medicine products. In the following section, we critically discuss the application of Bar-HRM methods in herbal medicine identification and provide an overview of developing a Bar-HRM assay.

### DNA Barcoding

DNA barcoding is a technique used to identify species based on a short, standardized fragment of the genome called “DNA barcode” (Hebert et al., 2003). This short sequence of nucleotides could be from an appropriate part of the chloroplast, mitochondrial, or nuclear genome and is used to identify organisms at the species level (Ryan, 2005; Miller, 2007). The mitochondrial coxI gene (COI) was suggested as a standard DNA barcode region for metazoans, and is now well established for most animal species identification (Hebert et al., 2003, 2004; Ward et al., 2005; Galimberti et al., 2012). However, the COI gene from plants have limited usefulness for identifying plant species due to the low amounts of variation in the genes, and shows intra-molecular recombination (Mower et al., 2007; Fazekas et al., 2008). Thus, screening for an analogous of COI gene in plants has focused on the nuclear and chloroplast genomes. Recent studies suggested that the internal transcribed spacer (ITS) of nuclear ribosomal DNA could be potential barcodes (Chase et al., 2005; Kress et al., 2005). In addition, several plastidial genes, such as the *rpo*C1, *rbc*L, *mat*K, *trn*H-*psb*A and *psb*K-*psb*I have also been proposed as barcode regions, because of their fast evolution rate (Kress et al., 2005; Fazekas et al., 2008). For example, Kress et al. (2005) compared 10 loci for authenticating closely related species in seven plant families and 99 species belonging to 88 genera in 53 families, and they reported that the *psb*A-*trn*H and ITS regions could be used as a pair of potential barcodes for identifying widely divergent angiosperm taxa (Kress et al., 2005). However, this pair failed to distinguish members of the order Cycadales (Sass et al., 2007). The Consortium for the Barcode of Life (CBOL) Plant Working Group (2009) suggested to combine *rbc*L and *mat*K as core-barcode regions, because of the straightforward recovery rate of *rbc*L, and the high resolution of *mat*K. Unfortunately, *mat*K is clearly lacking the feature of universal amplification (Dunning and Savolainen, 2010). In another study, two separate regions (*psb*A-*trn*H and *rbc*L) had significant discrimination efficiency and correctly discriminate 88% of 96 diverse species of 48 genera from 43 families (Kress and Erickson, 2007). Altogether, although no single plant marker has been found that works as well as the COI in animals, several markers such as ITS, *psb*A-*trn*H, *mat*K, *rbc*L, and *rpo*C1, have shown their superior qualities as core DNA barcodes.

Chen et al. (2010) compared seven candidate DNA barcodes (*psb*A-*trn*H, *mart*K, *rbc*L, *rpo*C1, *ycf*5, ITS2, and ITS) in more than 6,600 medicinal plant samples belonging to 4,800 species from 753 distinct genera. They found that the ITS2 of nuclear ribosomal DNA represents the most suitable region for DNA barcoding applications and that the rate of successful identification with ITS2 is 92.7% at the species level (Chen et al., 2010). Since then, the ITS2 region has been shown to be able to discriminate a wide range of medicinal plants (Sun et al., 2011; Pang et al., 2013; Zomuanpuii et al., 2013; Wu et al., 2015). Another three regions, *psb*A-*trn*H, *mat*K, and *rbc*L, have also been proposed as markers for barcoding medicinal plants (Liu et al., 2011; Mahadani and Ghosh, 2013; Nair et al., 2013). Although there is still debate on the effectiveness of DNA barcoding, this technology can achieve rapid, accurate, and automated identification of species from a diverse range and quality of raw materials. This addresses the difficulties involved in classifying herbal materials and promises to fuel a taxonomic renaissance in herbal identification.

### Bar-HRM is a Powerful Tool for Herbal Medicine Industry and Market

In markets, herbal products are commonly sold without packaging or labels, leading to a high risk of acquiring counterfeited, substituted and/or adulterated products. Species identification is critical to ensuring quality in the herbal medicine industry. Bar-HRM has been proven to be an effective tool for determining the origin and quality of raw materials and detecting adulterations (e.g., admixture with products from other species) in the herbal processed products chain (Kalivas et al., 2014; Tong et al., 2014; Buddhachat et al., 2015; Hu et al., 2015; Osathanunkul et al., 2015a,b; Singtonat and Osathanunkul, 2015; Costa et al., 2016). This new approach has its own advantage compare with previously employed methods, an advantage of performing Bar-HRM analysis is that the PCR amplification and HRM analysis are performed in the one completely “closed tube” run and the results are available for analysis at the end of the run. An overview of the Bar-HRM analysis developed in herbal medicine identification is provided in **Table 1.**

**Table 1:** Examples for application of Bar-HRM technology in herbal medicine identification.

Application	Target DNA region	Reference
Discrimination of 12 closely related *Croton* species	Internal transcribed spacer 1 (ITS1) and plastid DNA (*mat*K, *rbc*L, *rpo*C, and *trn*L)	Osathanunkul et al., 2015b
Taxonomic identification of *Sideritis* species growing in Greece	Internal transcribed spacer 2 (ITS2)	Kalivas et al., 2014
Differentiation of commercial *Panax notoginseng* from its adulterant species	The plastid DNA region *psb*A-*trn*H	Tong et al., 2014
Distinguish the Chinese herbs Mutong (*Akebia quinata*) and Chuanmutong (*Clematis armandii*) from Guanmutong (*Aristolochia manshuriensis*)	The plastid DNA region *psb*A-*trn*H	Hu et al., 2015
Evaluation of Three Medicinal Products Derived from Acanthaceae Species	*rbc*L chloroplast region	Osathanunkul et al., 2015a
Authentication of *Hypericum perforatum* and *Hypericum androsaemum* in herbal infusions	Nuclear ribosomal DNA region ITS1 and *mat*K chloroplast region	Costa et al., 2016
Detection of toxic *Crotalaria spectabilis* Roth. in *Thunbergia laurifolia* Lindl. herbal products	Four plastid DNA regions including *mat*K, *rbc*L, *rpo*C and *trn*L	Singtonat and Osathanunkul, 2015
Authenticity analyses of *Phyllanthus amarus* to control its quality for medicinal plant product	*trn*L and *rbc*L chloroplast regions	Buddhachat et al., 2015


Kalivas et al. (2014) developed an HRM based method coupled with DNA barcode ITS2 for verifying the identity of *Sideritis* species, evidencing Bar-HRM analysis was suitable for the identification of *Sideritis* species (Kalivas et al., 2014). This is the first report on the utilization of the HRM approach for rapid discrimination of *Sideritis* species and sparked a new line of research into possible identification for herbal medicine products. Since then, another research group has used HRM analysis method coupled with plastid DNA region *psb*A-*trn*H to distinguish traditional Chinese medicine *Panax notoginseng* from adulterant species (Tong et al., 2014), as well as the same method devoted to the identification of Chinese herbs Mutong (*Akebia quinata*), Chuanmutong (*Clematis armandii*), and Guanmutong (*Aristolochia manshuriensis*; Hu et al., 2015). These studies revealed that original species could be distinguished from adulterant species by differences in the melting curves.

Like DNA Barcoding method, the weakness of universal barcode markers was also existed in Bar-HRM analysis when identify closely related species, where genetic variability is limited. To overcome this weakness, Osathanunkul et al. (2015a) developed a minibarcoding method. By redesigning the *rbc*L primers, researchers were able to identify and authenticate three medicinal Acanthaceae species (*Acanthus ebracteatus*, *Andrographis paniculata*, and *Rhinacanthus nasutus*), demonstrating the reduced length of *rbc*L sequences could provided enough informative to identify closely related species (Osathanunkul et al., 2015a). In another study, Costa et al. (2016) pursued a very similar strategy to develop a Bar-HRM method for the authentication of *Hypericum perforatum* and *Hypericum androsaemum* in herbal infusions. They compared the effectiveness of ITS1 and *mat*K minibarcode for HRM analysis, and found that *mat*K can be used as an adequate mini-barcode for the differentiation of both species.

The identification of substitutions and admixtures in herbal products is one of the more interesting applications of Bar-HRM analysis. In many cases, commercial fraud involves adulteration, whether in the substitution of low-cost herbs for high-priced ones or the fraudulent labeling of herbal products. Adulteration also raises a number of concerns regarding health (e.g., toxic substances) and diet (e.g., nutritional value). Fraud control is therefore needed in order to support fair trade and protect consumer rights. In previous studies, DNA barcoding has proven successful for detecting substitution in herbal medicines (Chen et al., 2010; Liu et al., 2011; Mahadani and Ghosh, 2013; Nair et al., 2013). However, identifying the constituent species in a mixed herbal product has proven impossible unless preceded by a cloning approach, since the DNA extracted from these products yields contaminated PCR-amplification products (Galimberti et al., 2013). Bar-HRM may provide an effective approach to overcome this problem, allowing the successful identification of mixed herbal products. Buddhachat et al. (2015) and Singtonat and Osathanunkul (2015) demonstrated the use of Bar-HRM analysis to assess the amount of adulterant in herbal medicine products. Singtonat and Osathanunkul (2015) evaluated four DNA barcodes *mat*K, *rbc*L, *rpo*C, and *trn*L, and found that *rpo*C minibarcode coupled with HRM analysis was able to detect toxic herb *Crotalaria spectabilis* adulterants in *Thunbergia laurifolia* herbal products as low as 1% (**Figure 3D**). Another one study reported the same limit of detection for the contamination of *Phyllanthus amarus* with other *Phyllanthus* species. The authors also highlighted the rapid detection of Bar-HRM. In their study, the identity of an unknown contaminant in processed commercial herbal products can be verified within 2 h (Buddhachat et al., 2015).

These case studies and technical advancements clearly indicate that Bar-HRM is a sensitive, fast, cheap, and reliable method for identifying and tracking a wide range of raw and processed medicinal products, as well as for detecting adulteration or poisonous components potentially occurring in commercial frauds.

### Developing a Bar-HRM Assay for Herbal Material

Establishing a Bar-HRM assay for the identification of herbal material involves sample collection, verification of voucher herbarium specimens, DNA extraction, experiment optimization, HRM-PCR amplification, melting profile analysis, and finally, species identification and adulterant detection.

#### Sample Collection and Morphological Verification

It is crucial to be sure of the identity of the material used during the development of a Bar-HRM assay. At least three duplicate collections per species should be used; each collection (uniquely numbered) must include a small plant sample for DNA extraction and voucher herbarium specimens of the whole plant (ideally flowering or fruiting) and must be sourced from the same plant or population of plants. The collections should be accompanied by photos and detailed field notes describing any identifying characteristics not evident from the herbarium specimens. The samples must be in good condition (e.g., devoid of contamination). A detailed guide to making herbarium specimens can be found in the herbarium handbook (Bridson and Forman, 1992).

#### DNA Extraction

High-quality genomic DNA is an essential prerequisite for accurate identification using a Bar-HRM assay. DNA extraction from the herbal material must therefore be performed carefully and quickly, using good sterile technique to avoid DNA degradation and contamination between samples. Referred to related literatures, high quality DNA sample can be obtained by using modified CTAB method (Tel-zur et al., 1999; Chen et al., 2013, 2014). Reagents used in the method include the following: CTAB buffer containing 2% CTAB, 100 mM Tris-HCl, 5 mM EDTA, 710 mM NaCl, 350 mM Sorbitol, 0.1% Tris (2-carboxyethyl) phosphine hydrochloride and 1% 2-mercaptoethanol (added just before use); Washing buffer: 100 mM Tris-HCl, 5 mM EDTA, 5% glycerinum, 10% PEG8000, 0.1% Tris (2-carboxyethyl) phosphine hydrochloride; Extraction buffer: Chloroform:Isoamyl alcohol (24:1, vol/vol); polyvinylpyrrolidone (PVP). DNA extraction procedure consists of the following stages: (1) Harvest about 50–100 mg herbal material, clean the surface with 75% alcohol; (2) Mix the herbal material with 30 mg PVP, grind into a fine powder under liquid nitrogen; (3) Transfer the powder into a 2.0 ml centrifuge tube, add 1000 μl washing buffer, homogenize and keep on ice for 10 min; (4) Centrifuge the tube at 12,000 × *g* for 10 min at 4°C; (5) Remove and discard the supernatant, and then repeat steps 3–5; (6) Add 1000 μl CTAB buffer, mix and incubate at 65°C for 3 h with occasional mixing; (7) Centrifuge at 12,000 rpm for 10 min at 4°C, transfer supernatant to a new 2 ml tube; (8) Add an equal volume of extraction buffer to the tube and mix by inversion for 5 min; (9) Centrifuge at 12,000 rpm for 10 min at 4°C, transfer the top layer into a fresh tube; (10) Repeat steps 8–9; (11) Add an equal volume of cold absolute isopropanol and incubate for 1 h at –20°C; (12) Transfer the mixture into a spin column (Sangon, Shanghai, China) placed in a 2 ml collection tube, add 500 μl cold 70% ethanol, and centrifuge for 1 min at 10,000 rpm, discard the flow-through; (13) Add 500 μl absolute cold 100% ethanol, centrifuge for 3 min at 10,000 rpm; (14) Transfer the spin column to a new 1.5 ml centrifuge tube; (15) Add 50 μl ddH_2_O for DNA elution. The key points to consider in the DNA extraction of herb materials are summarized as following: (1) Because the herbal materials are usually harvested long before they are used, the surface of herbal material should be cleaned with 75% alcohol prior to being ground to a fine powder in liquid nitrogen for DNA extraction; (2) The high levels of polysaccharides and polyphenols in some herbal materials must be removed using polyvinylpyrrolidone (PVP) and β-mercaptoethanol during the early stages of DNA extraction; (3) In most cases, medicinal plant products are commonly sold in processed or modified forms such as dried material, tablets or powders, making its DNA degraded severely; thus, the dosage of plant starting material, PVP, and β-mercaptoethanol must be increased accordingly for DNA extraction.

#### Optimization

For successful analysis, the experimental conditions should be optimized for Bar-HRM. Small differences in melting curves can arise from factors such as the DNA quality and quantity, amplicon length, primer design, and cycling conditions. Optimize details was discussed above, under the Section “Key Factors Affecting HRM Assays.” Here, we summarize the key points as following: (1) All DNA samples should be extracted using the same DNA extraction method; the DNA sample should be diluted to a similar concentration in the same buffer. (2) The primer must be specific enough, and an optimal concentration is also required. (3) Adjustment of the annealing/extension time. Because shorter times result in incomplete amplicons, longer times can increase the possibility of mispriming and non-specific amplification. (4) The melting temperature should be adjusted to appropriate range according to the characteristic of the amplified products.

#### HRM-PCR Amplification

In theory, all of the plant DNA barcoding regions (i.e., ITS, *psb*A-*tr*nH, *rbc*L, *mat*K, *rpo*C, *trn*L, etc.) can used as barcodes for identifying herbal materials through Bar-HRM. For reference, we list the universal primers for the ITS2 barcode (S2F: 5′-ATG CGA TAC TTG GTG TGA AT-3′; S3R: 5′-GAC GCT TCT CCA GAC TAC AAT- 3′) and the *psb*A-*tr*nH barcode (PA: 5′-GTT ATG CAT GAA CGT ATG CTC-3′; TH: 5′-CGC GCA TGG TGG ATT CAC AAT CC-3′; Chen et al., 2010). In some cases, mini-barcode primers have been designed to distinguish samples from closely related species (Osathanunkul et al., 2015a,b; Costa et al., 2016). It is recommended that real-time PCR be conducted; amplification can then be monitored, and any troubleshooting that may be required is made easier. The reaction conditions and components used for PCR amplification can be found in the respective studies (Kalivas et al., 2014; Tong et al., 2014; Buddhachat et al., 2015; Hu et al., 2015; Osathanunkul et al., 2015a,b; Singtonat and Osathanunkul, 2015; Costa et al., 2016).

#### Melting Profile Analysis, Species Identification, and Adulteration Detection

Basic analysis of melting profile is described above, under the Section “HRM Data Analysis.” Difference curves, which accentuate the differences between individual melt curves, have been used to identify species. Test samples were defined by selecting an original representative sample for each species, and then a single reference-species melt curve was plotted as a baseline, providing improved visualization and allowing the separation of the melting curves of each species. Regardless of which species was used as the reference genotype, the position and shape of the difference curves of the other samples consistently allowed species identification (Kalivas et al., 2014). To detect the amount of adulterant in samples, standard purity curves were established by plotting the fluorescence level against the percentage of the adulterant (Buddhachat et al., 2015; Singtonat and Osathanunkul, 2015); the shape of the HRM curves indicates if the herbal product contains one or more adulterants.

## Conclusion

In recent years, the identification of species in herbal products has gained increased attention due to concerns about quality control and the satisfaction and safety of consumers. Recent advancements in molecular biology have led to the development of various criteria and methods to tackle this problem, one of the most remarkable being the rapid development of Bar-HRM technology. Although the application of Bar-HRM analysis in the authentication of herbal products is still in its initial stages, it shows great potential for identifying and tracking a wide range of raw and processed herbal medicine products, as well as detecting adulteration or poisonous contaminants in herbal products. With continued development and improvement, the Bar-HRM assay will become a significant resource for accurate species identification, monitoring, and quality control of herbal and other medicinal materials.

## Author Contributions

WS and J-jL wrote the manuscript. CX help to collect references articles. BZ help with language editing support. S-lC provided helpful comments on the article.

## Conflict of Interest Statement

The authors declare that the research was conducted in the absence of any commercial or financial relationships that could be construed as a potential conflict of interest.
